# Supplementary material for: Poly(vinylidene fluoride) Intestinal Sleeve Implants for the Treatment of Obesity and Type 2 Diabetes
Source: Polymers (Basel). 2022 May 27;14(11):2178. doi: 10.3390/polym14112178 (PMC9183030; doi:10.3390/polym14112178)
Supplement: Supplementary file 1 [file polymers-14-02178-s001.zip › polymers-1717195-supplementary.pdf]

polymers
MDPI

Supplementary Information

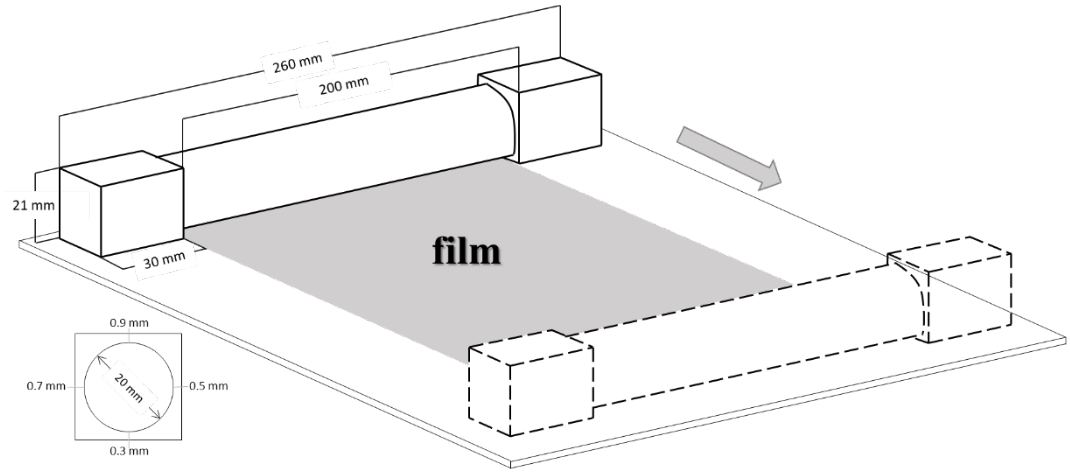

Figure S1. Schematic diagram of the self-designed film coater.

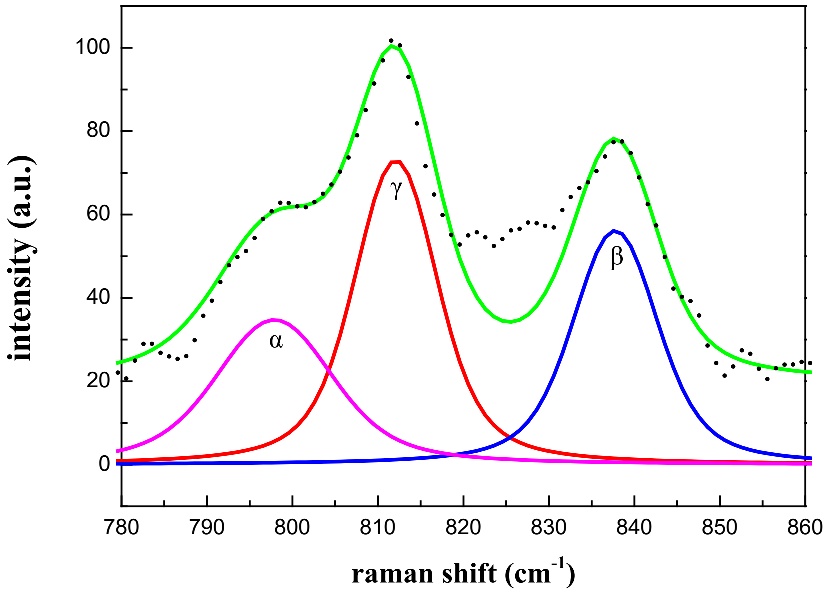

Figure S2. Raman spectroscopy peak splitting schematic diagram.

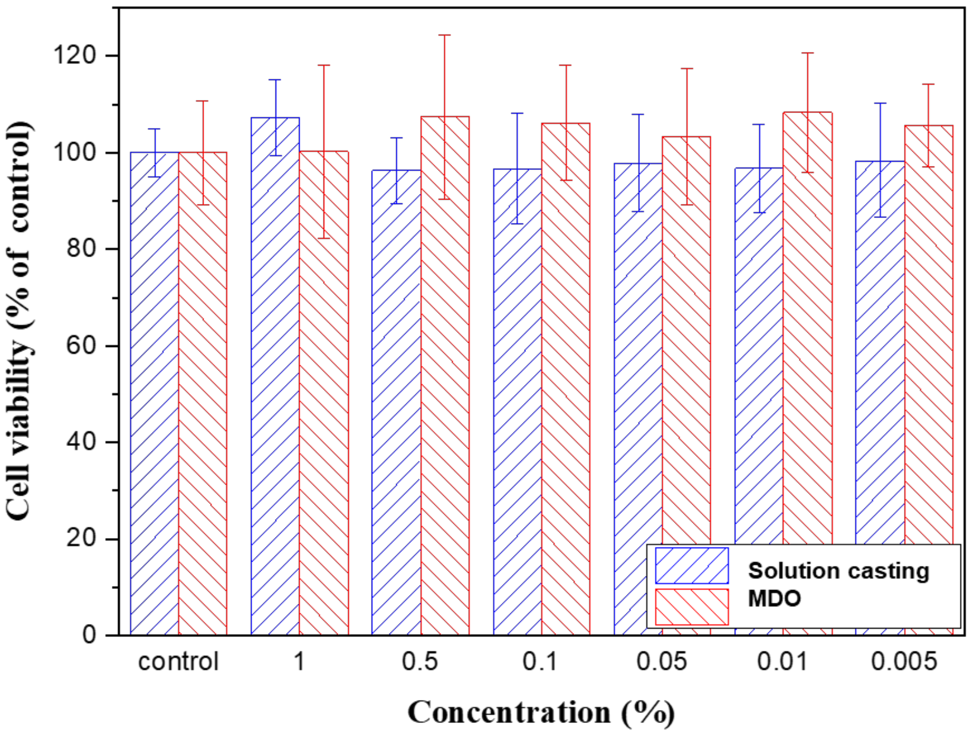

Figure S3. Cytotoxicity test of PVDF with different process methods.

Table S1. Reference of PVDF FTIR characteristic peak.

Wavenumbers (cm^{-1})	Vibration mode	Phase
616	CF_2 bending	α
763	CF_2 In-plane bending/rocking	α
798	CH_2 rocking	α
810	CH_2 wagging vibration	γ
839	CH_2 out of plane wagging CF_2 bending	β
975	CH_2 twisting	α
1180	CF stretching vibration	α
1209		α
1234		γ
1275		β
1382	CH_2 rocking	α
1431		β

Table S2. Reference of PVDF Raman characteristic peak.

**Raman Shift (cm^{-1})**	**Vibration mode**	**Phase**
485		γ
513	CF_2 Scissoring	β
610	CF_2 Scissoring	α
795	CH_2 Rocking	α
812	CH_2 Wagging	γ
840	CH_2 Rocking	β
881	CC Vibration Symmetric	α,β
1076	CC Vibration Asymmetric	
1171	CH_2 Wagging	
1265	CC Vibration Symmetric<br>CCC Scissoring	
